# An Overview of Next-Generation Androgen Receptor-Targeted Therapeutics in Development for the Treatment of Prostate Cancer

**DOI:** 10.3390/ijms22042124

**Published:** 2021-02-20

**Authors:** Michael L. Mohler, Arunima Sikdar, Suriyan Ponnusamy, Dong-Jin Hwang, Yali He, Duane D. Miller, Ramesh Narayanan

**Affiliations:** 1 Department of Pharmaceutical Sciences, College of Pharmacy, University of Tennessee Health Science Center, Memphis, TN 38163, USA; michael_mohler@yahoo.com (M.L.M.); dhwang@uthsc.edu (D.-J.H.); yhe33@uthsc.edu (Y.H.); dmiller@uthsc.edu (D.D.M.); 2 Department of Medicine, College of Medicine, University of Tennessee Health Science Center, Memphis, TN 38103, USA; asikdar@uthsc.edu (A.S.); tponnusa@uthsc.edu (S.P.)

**Keywords:** prostate cancer, castration-resistant prostate cancer (CRPC), androgen receptor (AR), selective AR degraders (SARD), non-canonical

## Abstract

Traditional endocrine therapy for prostate cancer (PCa) has been directed at suppression of the androgen receptor (AR) signaling axis since Huggins et al. discovered that diethylstilbestrol (DES; an estrogen) produced chemical castration and PCa tumor regression. Androgen deprivation therapy (ADT) still remains the first-line PCa therapy. Insufficiency of ADT over time leads to castration-resistant PCa (CRPC) in which the AR axis is still active, despite castrate levels of circulating androgens. Despite the approval and use of multiple generations of competitive AR antagonists (antiandrogens), antiandrogen resistance emerges rapidly in CRPC due to several mechanisms, mostly converging in the AR axis. Recent evidence from multiple groups have defined noncompetitive or noncanonical direct binding sites on AR that can be targeted to inhibit the AR axis. This review discusses new developments in the PCa treatment paradigm that includes the next-generation molecules to noncanonical sites, proteolysis targeting chimera (PROTAC), or noncanonical N-terminal domain (NTD)-binding of selective AR degraders (SARDs). A few lead compounds targeting each of these novel noncanonical sites or with SARD activity are discussed. Many of these ligands are still in preclinical development, and a few early clinical leads have emerged, but successful late-stage clinical data are still lacking. The breadth and diversity of targets provide hope that optimized noncanonical inhibitors and/or SARDs will be able to overcome antiandrogen-resistant CRPC.

## 1. Background

Prostate cancer (PCa) is the most common non-cutaneous cancer among men. Although it is slow growing, often with no symptoms and being highly treatable in early stages, the American Cancer Society predicts in 2020 that there will be around 33,000 deaths due to PCa [1]. Most of these deaths occur from advanced forms of PCa called castration-resistant PCa (CRPC). CRPC can be metastatic (mCRPC) or non-metastatic CRPC (nmCRPC), which grows aggressively and results in shorter overall survival (OS). Treatment for early PCa includes attempts at curative therapies such surgery, cryotherapy, proton therapy, and/or radiation therapy, sometimes followed by adjuvant pharmacotherapy in high-risk patients such as chemotherapy, androgen-deprivation therapy (ADT), and/or hormone therapies. However, when curative and adjuvant therapies fail, as judged by rising prostate-specific antigen (PSA) levels or metastasis, currently available hormone therapies (various forms of indirect or direct androgen receptor (AR) antagonism) are employed to delay progression of (but cannot cure) PCa.

**Figure 1:**
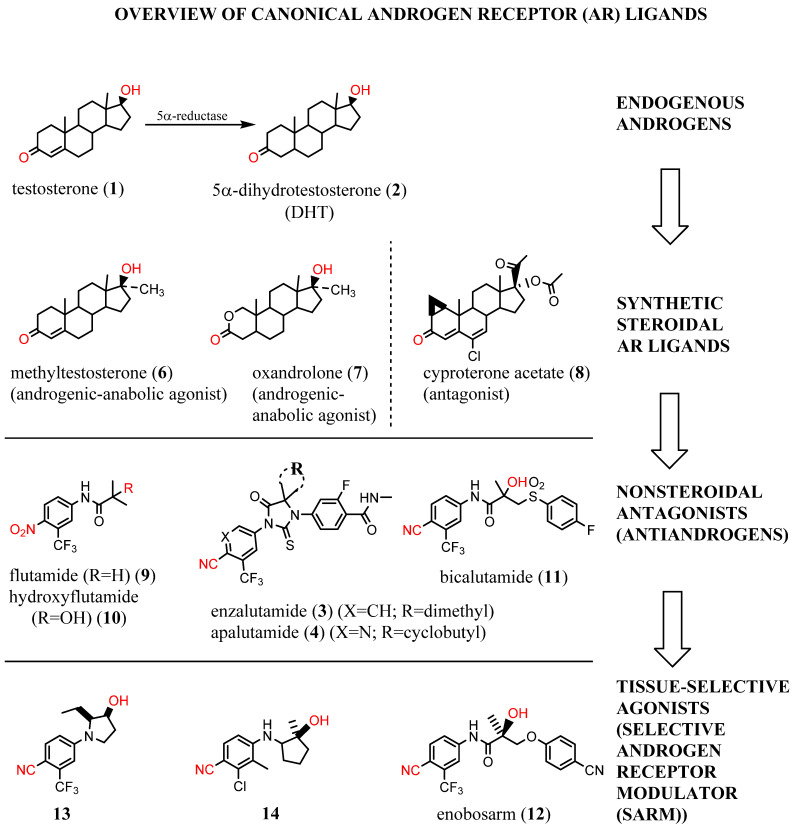
Overview of canonical androgen receptor (AR) ligands.

More than 90% of the early stage PCas are primarily dependent on the androgens, namely, testosterone (**1**) and dihydrotestosterone (DHT) (**2**) (Figure 1), for growth [2]. Androgenic hormones bind to the AR, a member of the hormone receptor family of ligand-activated transcription factors, in the cytoplasm and promote translocation of the AR into the nucleus. In the nucleus, the AR binds to DNA regions called androgen response elements (AREs), which are palindromic sequences, recruits cofactors, and general transcription machinery, and that promote transcription and translation of the target genes [3]. AR is the primary therapeutic target in PCa and CRPC [4,5]. Although the early stage PCa is AR-driven in more than 90% of the cases, this percentage decreases in later stages of CRPC and mCRPC, where still around 70–80% of the cases require AR for growth [2,6,7]. Still, this is considered as a high percentage reliant on a single therapeutic target, and hence the preponderance of therapeutic modalities target antagonism of the AR axis.

Resistance to ADT such as gonadotropin-releasing hormone agonist or antagonist (or alternatively surgical castration) results in progression to CRPC. Subsequent or concomitant resistance to AR blocking agents such as the androgen synthesis inhibitor; abiraterone; and/or antiandrogens such enzalutamide (**3**), apalutamide (**4**), or darolutamide is inevitable. Several mechanisms have been attributed to these resistances including overexpression of the AR, mutations in the AR ligand-binding domain (LBD), loss of AR (neuroendocrine PCa), constitutively active AR splice variants (AR-SVs), increase in intratumoral hormonal synthesis, and activation of growth factor pathways [8]. Over time, as the AR milieu present in the PCa becomes complex and heterogeneous, patients become refractory to AR blocking agents [9]. The AR-SVs have emerged as critical players in the development and progression of mCRPCs. Among AR-SVs identified to date, AR-V7, also known as AR3, is one of the most abundant and frequently found forms in both PCa cell lines and human prostate tumors [10]. Notably, the lack of LBD indicates that all Food and Drug Administration (FDA)-approved AR blocking agents will have no efficacy in inhibiting AR-SVs. Genomic events leading to AR-SV expression could act as novel biomarkers of disease progression that may guide the optimal use of current and next-generation AR-targeted therapy [9].

Endocrine therapy-resistant PCa cells generated by chronic treatment with **3** or abiraterone showed enhanced AR-V7 protein expression [11,12]. Knockdown of AR-V7 by small interfering RNA (siRNA) in abiraterone-resistant CWR22Rv1 and C4-2B enzalutamide (MDV3100)-resistant (MDVR) cells restored their sensitivity to abiraterone, indicating that AR-V7 is involved in abiraterone resistance. Moreover, an FDA-approved anthelminthic drug, niclosamide (**5**) [13], has been previously identified as a potent inhibitor of AR-V7, re-sensitizes resistant cells to **3** or abiraterone treatment in vitro and in vivo [13,14].


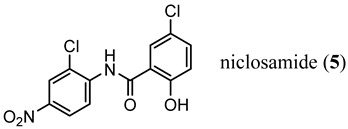


## 2. Structural Basis for Antagonist Development

Elucidation of the crystal structures of the AR DNA binding domain (DBD) and the LBD provides a new framework for understanding the functions of this receptor and has led to the development of rational drug design for the treatment of prostate cancer. Despite the lack of any specific structural characterization, N-terminal domain (NTD) inhibitors are also in preclinical development.

Early ligand-based drug design was based on modification of structure of testosterone, the most prevalent endogenous androgen. Starting in the 1930s, many steroidal agonists such as methyltestosterone (**6**) and oxandrolone (**7**) and steroidal antagonists such as cyproterone acetate (**8**) were approved.

**Figure 2:**
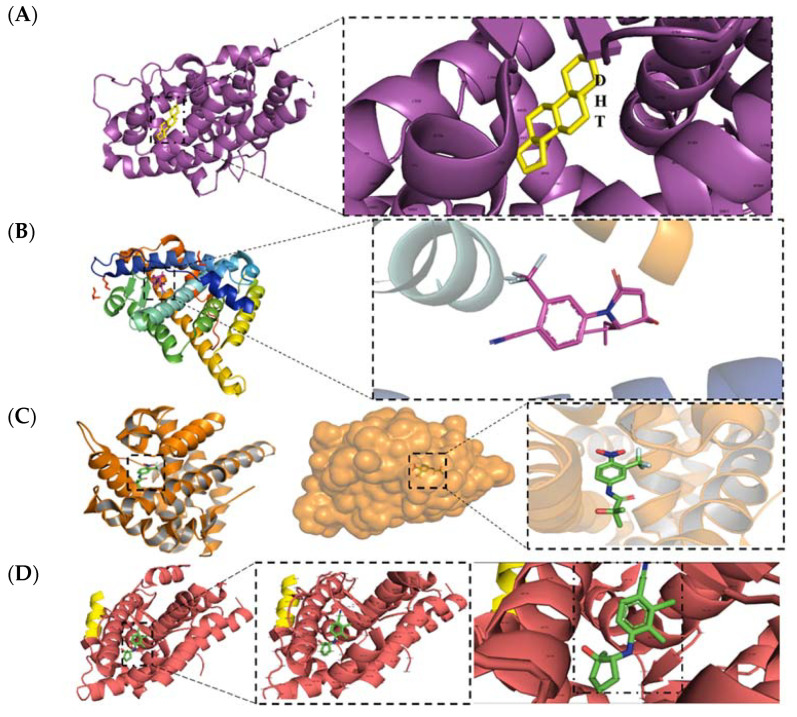
Conserved binding model for canonical ligands of the AR. Panel **A**: Cartoon model of a crystal structure of dihydrotestosterone (DHT) (**2**) bound to the ligand-binding domain (LBD) (Protein Database (PDB) ID: 4OEA [23]). Panel **B**: Cartoon model of a crystal structure of a selective androgen receptor modulator (SARM) **13** (PDB ID: 5V8Q [24]. Panel **C**: Cartoon model of a crystal structure of hydroxyflutamide (**10**) bound to the T877A mutant AR LBD (PDB ID: 2AX6 [25]). Panel **D**: Cartoon model of a crystal structure of a SARM **14** bound to the LBD (PDB ID: 5CJ6 [26]).

Unfortunately, cross-reactivity with other nuclear hormone receptors, liver toxicity, and inability to separate anabolic (muscle/bone building) from androgenic (growth of primary and accessory sexual organs) activity has limited the utility of steroidal ligands. In the 1960s, it was discovered that 5α-reductase converted testosterone (**1**) to DHT (**2**) as a local amplification of the AR axis in certain tissues such as the skin and prostate [15]. Not until 2001 was the first ligand co-crystal structure of AR elucidated. This wild-type androgen receptor (wtAR)–DHT (Figure 2A) structure was able to elucidate the binding mode for **2** and other the steroidal AR ligands [16]. The endogenous steroid **2** bound to the AR via an internalized, i.e., not solvent-exposed, hormone binding pocket (HBP) in the C-terminal LBD.

The first nonsteroidal AR ligand, flutamide (**9**), was discovered as an AR antagonist in the 1980s [17]. Flutamide and its active metabolite, hydroxyflutamide (**10**), were the first in a series of agents termed as antiandrogens, including bicalutamide (**11**), the first diphenyl nonsteroidal AR ligand [17,18]. In the 1990s, the first nonsteoridal AR agonists were discovered as derivatives of **11** in which the sulfonamide of **11** was replaced with thioethers (not shown); however, the thioethers lacked in vivo agonist activity [19]. Conversion of the thioethers to ethers resulted in the tissue-selective AR modulators (SARMs) such as enobosarm (**12**), which acted as agonists in anabolic tissues such as the musculoskeletal system (as reflected by the levator ani muscle) and weak partial agonists (or antagonists) of the anabolic tissues such as ventral prostate and seminal vesicles [20,21,22].

Modern structural biology allowed many AR agonist co-crystal structures to be solved, such as **13** and **14** (Figure 2B,D), which are shown as recent representative examples [24,26]. Unfortunately, no antiandrogen–wtAR co-crystal structure exists yet, limiting the potential of structure-based antiandrogen design. Antiandrogen resistance is frequently caused by mutations in AR-LBD (mtAR) in such a manner that the steric bulk of the antiandrogen (see W741L for bicalutamide (**11**) or F876L for **3** or **4**), which destabilizes AR, is accomodated by mutation to a smaller amino acid [25]. These antiandrogen–mtAR complexes activate the AR axis, leading to a type of mutational resistance referred to an agonist switch or escape mutation [27]. Co-crystal structures of antiandrogens bound to their agonist switch mutation have been published, and the T877A and hydroxyflutamide (**10**) co-crystal structure serves as a representative escape mutation (Figure 2C) [25].

## 3. Need for AR-Directed Therapy Beyond Canonical Antiandrogens

As described above, the discovery of antiandrogens that compete for binding with testosterone (1) and DHT (2) to the HBP of the LBD continues to be productive, but antiandrogen resistance via agonist switch and AR truncation (e.g., AR-V7) mutations limits their duration of efficacy. Correspondingly, innovative new approaches to provide hormone therapy in CRPC are needed. This review seeks to briefly outline a variety of novel small molecule ligands with AR antagonist activity and/or noncanonical AR binding sites with the goal of stimulating interest in further discovery of noncanonical ligands that may overcome known mechanisms of FDA-approved endocrine therapies.

### 3.1. LBD-Targeted SARDs

An emerging alternative approach to relying on competitive binding of the HBP to prevent endogenous androgen action is to downregulate (decrease expression or destroy (destabilize or proteolyze)) the AR, thereby eliminating AR-mediated signaling [28]. Selective AR downregulators or degraders (SARDs) have been discovered, which bind to the LBD (discussed here) or the NTD (vide infra). In general, direct binding to AR is preferred to provide AR specificity, and degradation (protein destruction) is preferred to downregulation. ASC-J9 (15) was an early downregulator that was not demonstrated to bind to AR but lowered AR levels and produced antiproliferative activity in C4-2 and DU145 (AR-negative) PCa cells with artificially expressed F876L [29], an escape mutant conferring **3** or **4** resistance. Another small molecule downregulator is AZD3514 (**16**), which was demonstrated to bind to AR LBD and downregulate AR. In two phase I clinical trials, moderate anti-tumor activity was observed in CRPC patients as judged by significant PSA declines, but the drug was not well tolerated. Although development was discontinued, **16** provided a rationale for future development of SARD compounds [30].


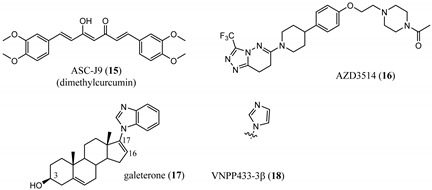


Galeterone (**17**) [31] is a steroidal antiandrogen developed for the treatment of CRPC. It possesses a unique combination of three separate biochemical mechanisms of action, namely, acting as an antagonist that binds competitively to AR LBD, inhibiting CYP17A1 with selectivity for 17,20-lyase activity over 17α-hydroxylase activity of CYP17A1, and acting as an AR degrader that markedly enhances AR degradation [32,33,34].

Galeterone (**17**) (also known as TOK-001 or VN/124-1) was developed by Tokai Pharmaceuticals and tested in a phase III clinical trial (ARMOR3-SV) in 2016 for AR-V7-positive mCRPC [32,35]. The studies revealed that **17** has poor oral bioavailability, which undoubtedly contributed to its clinical failure in phase III. The multi-targeted lyase inhibitor and direct AR antagonist/SARD 17 is a novel approach that is still in clinical development by the University of Maryland, Baltimore (NCT04098081), with a next-generation lead **18** still in preclinical development [36].

### 3.2. Proteolysis-Targeting Chimeras (PROTACs) as Nonsteroidal AR Degraders

**Figure 3:**
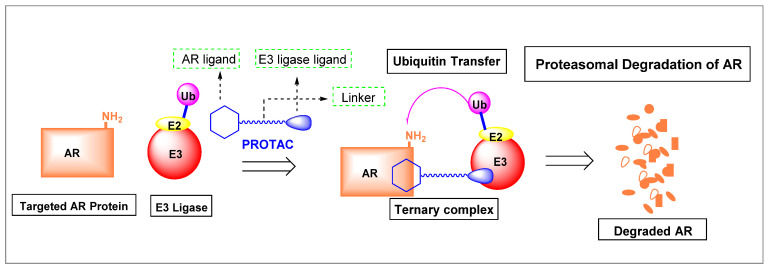
Mechanism of action of AR-directed proteolysis-targeting chimeras (PROTACs). The AR ligand warhead binds AR and the E3 ligase warhead binds to E3 ligase, forming a ternary complex. The ternary complexes allow for efficient catalysis of the transfer of ubiquitin (Ub) to lysine (NH_2_) residues on the AR, targeting AR to be destroyed by the proteosome.

PROTACs targeted to AR are heterobifunctional small molecules that consist of a central linker and two warheads, one warhead is based on a known AR ligand to bind AR and the other recruits an E3 ligase, as shown in Figure 3. Although activity is through HBP binding, PROTACs provide targeted AR protein degradation at very high potency, unlike traditional canonical antiandrogens. PROTACs bring E3 ubiquitin ligase enzyme to AR to form a ternary complex, which results in very efficient ubiquitination of AR, causing AR degradation by the proteasome (Figure 3).

Since first proposed by Crews et al. [37], PROTAC technology has employed several AR ligands to target the AR. These AR ligands were based on known antagonists or SARMs but resulted in AR degradation rather than antagonizing the AR axis by competitive binding alone. A variety of E3 ligases and linkers have been employed and tested. Correspondingly, substantial effort is required to optimize PROTACs by synthesizing the various possible combinations of AR ligands, linkers, and E3 ligases, as summarized below.

**Figure 4:**
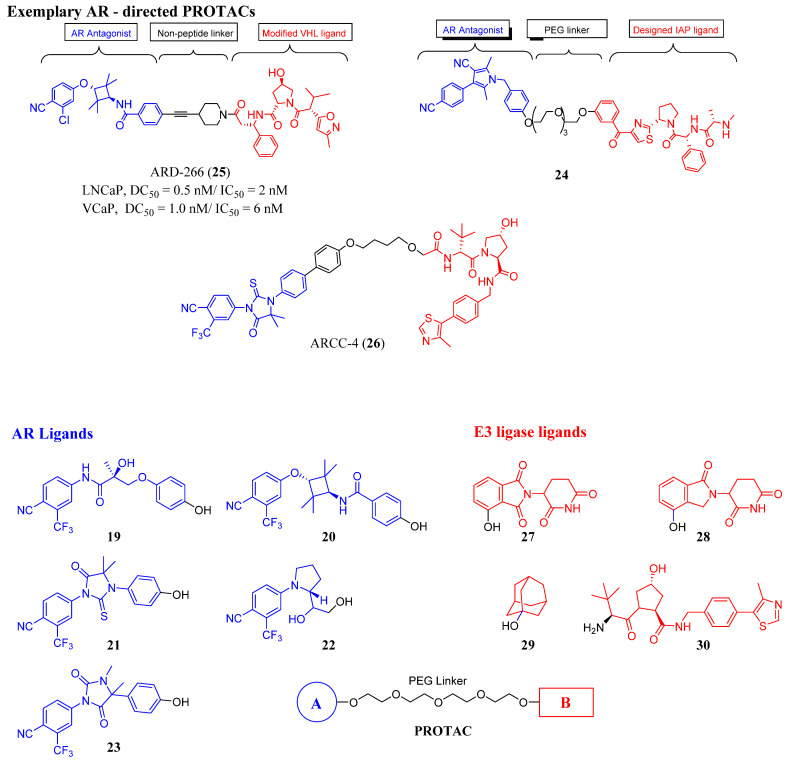
Exemplary AR-directed PROTACs and examples of AR ligands and E3 ligase ligands [39,40,41,42,43,44].

Nonsteroidal AR ligands offer greater nuclear receptor selectivity as well as being relatively amenable to synthetic modification. Examples of nonsteroidal AR warheads include **19**-**23**, as shown in Figure 4. Research found that **19** is derived from the SARM enobosarm (**12**) [38], whereas **21** is derived from enzalutamide (**3**).

Early PROTACs possessed peptide-based linkers, but non-peptide-based linkers are preferred, or various lengths of polymers such as polyethylene glycol (PEG)-based (e.g., see in 24) to connect the warheads. The complexity of PROTAC design has increased as more complex linker segments are now commonly employed (see black atoms in ARD-266 (25) and ARCC-4 (26)).

A wide variety of E3 ligase warheads has been explored. A representative sample of four different E3 ligases are shown in AR-targeted PROTACs in Figure 4, namely, MDM2 ligands based on nutlin, cIAP1 ligands based on bestatin esters, Von Hippel–Lindau (VHL) ligands based on the hydroxyproline structure found in HIF1a, and cereblon (CRBN) ligands based on thalidomide (Figure 4) [39].

Many AR-degrading PROTACs exist, however, ARCC-4 (**26**) is shown here as an exemplary SARD [40,45,46]. ARCC-4 (**26**) in Figure 4 utilizes **3** as the AR ligand and VHL as the ubiquitin ligase, thereby targeting AR to the protease for degradation. ARCC-4 (**26**) was able to degrade AR in VCaP cells (resistance mechanisms include AR amplification, AR-V7 expression, and TMPRSS2–ERG (a fusion of TMPRSS2 (transmembrane protease, serine 2) to ETS-related gene (ERG)) translocation) at 5 nM with maximal degradation of 98%. ARCC-4 (**26**) was able to degrade wtAR and a variety of escape mutants with nanomolar potencies including F876L (confers resistance to **3** and **4**) [47], W741L (confers resistance to bicalutamide (**11**)) [48], M896V (confers resistance to **11**) [49], T877A (confers resistance to flutamide (**9**), glucocorticoids, and progesterone) [50], H874Y (confers resistance to **9**, glucocorticoids, and progesterone) [50,51], and L702H (glucocorticoids) [52]. As illustrated by **26**, PROTACs tend to be large compounds that do not comply with standard rules for oral bioavailability, and thus attaining this broad scope of antiandrogen activity in vivo may be difficult. Another disadvantage with PROTACs, assuming full oral bioavailability, is the limitation of requiring an LBD (canonical AR ligands have been employed thus far) to destroy the AR, indicating that AR-SVs may not be destroyed by these PROTACs. Lastly, PROTACs do not eliminate the possibility of PR- or GR-mediated AR-axis signaling, referred to as AR bypass resistance. A reportedly orally bioavailable SARD whose structure is undisclosed, ARV110, developed by Arvinas, was initiated in phase I trials in 2019 in a late-stage mCRPC patient population, and recently released an interim data update (https://ir.arvinas.com/news-releases/news-release-details/arvinas-releases-interim-clinical-data-further-demonstrating; accessed January 2021). Dose escalation of ARV110 produced PSA responses at 280 mg, and at 420 mg attained sufficient exposure to have antitumor effects as judged by analogy to preclinical models. Two of five patients (40%) with H874 or T877 mutations had PSA reductions >50% compared to 2 of 15 wtAR patients, supporting testing of a molecularly defined late-line patient population. Additionally, Arvinas is considering testing in a less pre-treated population. This data established the proof-of-concept that it is possible to have clinical efficacy with an orally active AR-directed PROTAC; however, the high dose (420 mg) to achieve efficacious exposures in contrast to the low nanomolar preclinical potencies suggests that pharmacokinetic (PK) properties could be improved.

### 3.3. Summary of Hormone Binding Pocket (HPB)-Targeted Antiandrogens

Two generations of (competitive) antiandrogens have been approved, and with each new approval, the scope of antiandrogen activities was expanded and/or antiandrogen potencies improved. For example, flutamide (**9**) was less potent than bicalutamide (**11**), and both were susceptible to resistance due to AR overexpression. Enzalutamide (**3**) and apalutamide (**4**) could overcome AR overexpression and demonstrated the novel ability to inhibit nuclear translocation of the AR; however, all these antiandrogens were susceptible to escape mutations [47,53]. Darolutamide also inhibited translocation and overcame AR overexpression, but additionally was a more potent inhibitor than first- and second-generation antiandrogens and was a pan-antagonist of escape mutations [47,53]. Similarly, PROTACs are also able to overcome AR overexpression and have demonstrated perhaps the broadest scope of pan-antagonism of HBP-dependent antiandrogens; however, PROTACs are in early stages of clinical testing with limited efficacy reported thus far. Despite the progressively broadening scope of antiandrogen activities, made possible with HBP-directed antiandrogens, none of these approaches are able to address the problem of inhibiting AR-SV-dependent PCa growth.

## 4. Noncompetitive Antiandrogens

Developing nonconventional AR-modulating agents targeting noncanonical binding sites is likely to be a promising future approach to develop multiple and synergistic strategies [54].

LBD and DBD crystal structures facilitated the discovery of solvent-exposed regions on the surface of AR that are suitable for the design of non-competitive ligands. Ligands of the NTD have also been reported, despite the intrinsically disordered nature of the NTD. All noncompetitive ligands thus far have been inhibitors and are expected to have unique properties when compared to the FDA-approved competitive inhibitors. Noncompetitive antagonists are reported for the following noncanonical sites: (1) activating function (AF)-2 and (2) binding function-3 (BF-3) sites that reside in the LBD, DBD sites include (3) the DNA recognition helix [55] and (4) the DBD/dimerization region (not discussed here, but reported) [56,57], and (5) AF-1 inhibitors that bind to and inhibit the NTD.

### 4.1. Noncanonical (Noncompetitive) LBD Ligands

The LBD consists of the C-terminal residues from 676 to 919 and, in addition to the internal HBP, also possesses the AF-2 function, which forms an external binding surface for interactions with co-regulatory proteins recruited upon agonist binding. The noncanonical LBD inhibitors have had weak antiandrogen potencies but nonetheless provide novel ways to modulate the AR axis by directly blocking (AF-2 inhibitors) or modulating (BF-3 inhibitors) the protein–protein interactions that occur between AF-2 and its cofactors [58]. Direct AF-2 inhibitors mimic the LXXLL or F/WXXLF consensus-binding motifs of cofactors and thereby bind competitively with cofactors for occupancy of the AF-2 site. Initially peptides were discovered and tested as AF-2 inhibitors. Phage display studies revealed that mtAR and wtAR bound to similar peptidomimetic inhibitors of AF-2, including BUD31 (structure not shown), which contains an Fxx(F/H/L/W/Y)Y motif cluster with tyrosine (Y) in their respective position [23]. Thus, structural analyses of the AR–LBD–BUD31 complex revealed the formation of an extra hydrogen bond between the specific Y residue of the peptide and AF-2. A combination of peptide screening and X-ray structure analysis may serve as a new strategy for developing AR antagonists that simultaneously stop both wild-type and mutated AR function.

IMB-A6 (**31**), an exemplary small molecule inhibitor of the AF-2 site, was discovered on virtual screening for ligands that bind to the AF-2 site. IMB-A6 (**31**) demonstrated the ability to inhibit binding of co-factor PELP-1 (proline-glutamic acid- and leucine-rich peptide) at 10 µM range (noncompetitive with HBP ligands) [59]. Another novel allosteric pocket on the LBD, one that is potentially amenable to pharmacological manipulation, is named binding function-3 (BF-3). The BF-3 pocket is adjacent to the AF-2 pocket, and small molecule ligands of BF-3 regulate which co-regulators bind to AF-2, resulting in a modulation of AR activity similar to SARM but not through HBP. An exemplary BF-3 binding compound, MEPB (**32**), was shown unequivocally by X-ray crystallography to bind the BF-3 domain [60]. MEPB (**32**) was recently tested in a model of spinobulbar muscular atrophy (SBMA), a neurodegenerative disorder in males characterized by expanded length CAG trinucleotide repeat (long poly-Q tract). In SBMA, the long poly-Q tract results in toxic neurodegenerative effects upon androgen stimulation of defective AR. MEPB (**32**) caused increased recruitment of co-repressors such as nuclear receptor corepressor (NCoR) to the AF-2 domain and ameliorated neuronal loss, neurogenic atrophy, and testicular atrophy in a mouse model of SMBA, validating AF-2 modulation as a potent androgen-sparing strategy for SBMA therapy [61].


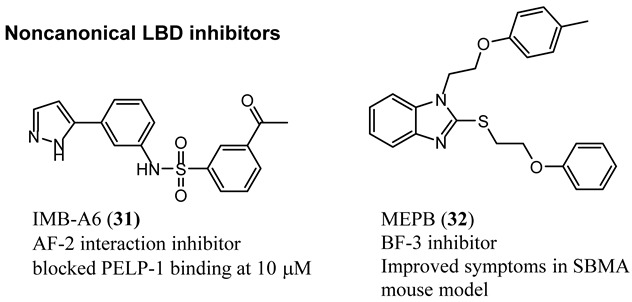


### 4.2. Noncanonical DBD Antiandrogens

A theoretical advantage of targeting the DBD is that potent inhibition of the DBD to DNA interaction would effectively abrogate transcriptional activation and would be unaffected by LBD point mutations (e.g., escape mutants) or LBD truncations (e.g., AR-SVs). A theoretical disadvantage is that the DBD is the most conserved domain between steroid receptors, and thus obtaining steroid receptor selectivity might be challenging.

Characterizing DBD by X-ray crystallography provided structures that facilitated the discovery of surface-exposed regions of the AR that might serve as a noncanonical ligand binding site. In silico virtual screening of residues S579 to K610 identified candidate molecules that were effective at abolishing transcription in LNCaP cells [55]. Screening of the candidates demonstrated activity for a series of compounds consisting of a system of three rings directly connected by single bonds, with the middle ring being heterocyclic and most often a thiazole. Synthetic expansion explored substituted aryl A-rings with a thiazole B-ring and a morpholino C-ring. A lead that emerged from this effort was VPC14449 (**33**), which suppressed PSA expression comparable to enzalutamide (**3**) (0.17 μM vs. 0.12 μM) in LNCaP cells [55].


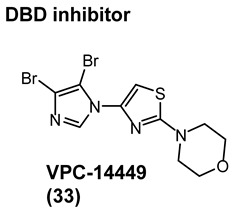


In an artificial in vitro transcriptional activation assay, **33** and **3** again displayed similar potency (0.340 μM vs. 0.314 μM) [62]. To validate the DBD binding site, the researchers prepared point mutations Y594D and Q592D, which were found to be inhibited by **3** but not by **33**, supporting the proposed binding site. VPC14449 (**33**) fully inhibited wtAR at 5 μM but only partially affected estrogen receptor at 15 μM and did not affect glucocorticord receptor or progesterone receptor at <25 µM, demonstrating surprising nuclear receptor selectivity (similar to **3**). On the basis of this in vitro characterization, the researchers deemed **33** to be a useful prototypical DBD inhibitor and was tested in models of CPRC [63] such LNCaP (T877A), C4-2 (T877A), MR49F and MR49C (F876L_T877A), and 22RV1 (H874C and overexpressed AR-V7).

### 4.3. Noncanonical NTD Antiandrogens

Two separate groups are developing antiandrogens that bind to the AF-1 region. Mutational analysis has revealed two regions in the NTD, encoded by amino acid residues 141-338 (transcription activation unit (Tau)-1) and 380-529 (Tau-5), to be essential for this transcriptional activation, being termed as the AF-1 [64]. AR has the capacity to use different regions in the N-terminal domain (residues 1-450) as transcription activation units (TAUs) [65]. The inhibitors below have been reported as blocking tau-5 (EPI-506 (**34**)) [66] and tau-1 (UT-155 (**35**)) [67]. Deletions of the AF-1 region rendered AR transcriptionally inactive [68]. In contrast, deletion of LBD caused constitutive activation, suggesting that, in the absence of bound androgen, the LBD exerts an inhibitory effect on AR. This helps to rationalize the selective advantage of expressing AR-SVs in tumor cells subjected to LBD competitive antagonists. Collectively, this provides a rationale for inhibiting the function of AF-1 in heavily pre-treated CRPC populations that are expressing high levels of a heterogenous mixture of wtAR, AR-SV, and escape mutations. Unlike DBD and LBD, which are intrinsically ordered (globular) domains, the NTD is intrinsically disordered, confounding efforts to use structure-based drug design [69]. Binding to coregulatory binding partners is postulated to induce folding of the NTD into α-helical conformations in order to maintain affinity, and similarly, small molecule ligands may also induce or stabilize secondary or tertiary structure within the otherwise disordered domain [69].

### 4.4. AF-1 Inhibitors Derived from Natural Products

A new generation of androgen receptor antagonists including **34** was discovered in 2010 in vitro assays. Unlike bicalutamide (**11**), these antagonists had noncompetitive activity as judged by the lack of a right shift with increasing concentrations of an agonist such as R1881 and were not able to displace fluoromone from the HBP (i.e., a fluorescent hormone that binds to the HBP) [70], supporting a noncanonical binding site. Preclinical investigation revealed that **38** was the active component of a mixture of hydroxyl group epimers, irreversibly bound to the N-terminal domain of the AR via the 20*S* chlorohydrin [71], and was able to inhibit androgen-dependent genes in cells expressing only AR-SV (v567es). The irreversibility of the interaction was postulated as a basis for inhibiting the intrinsically disordered NTD. In VCaP tumors overexpressing full-length (FL) AR and expressing AR-V7, **38** inhibited AR-V7-dependent genes, i.e., genes shown to be uniquely upregulated by AR-V7 and not AR-FL [72]. Although micromolar levels were required to observe effects in vitro, the triacetate prodrug **34** was tested in patients that had failed enzalutamide (**3**) or abiraterone therapy, finding only a reduction of PSA by <30% for a short period of time. Further, very high doses of up to 3.6 g were required. Due to the poor pharmacokinetics, the trial was stopped. This was interpreted as a proof-of-principle but insufficient potency and drug exposure (i.e., rapid metabolism) to inhibit the heterogenous and resistant ARs present in this difficult-to-treat patient population, and new agents were prepared.

Compounds from this template with 20-fold greater potency and improved pharmacokinetics have been reported [73]. For example, EPI-7170 (**39**), a semi-synthetic sulfonamide NTD antiandrogen derived from the bisphenol A (**37**) nucleus, was reported to provide synergistic activity in AR-V7 PCa when combined with the canonical antiandrogen **3**. A very recent new drug, EPI-7386 (structure unknown), has been characterized preclinically in VCaP and several other xenografts. EPI-7386 demonstrated a significant inhibition of VCaP tumors in castrated mice with a tumor growth inhibition of close to 100%. ESSA Pharma reported the compound to have entered clinical trials (NCT04421222) for PCa. The reported irreversibly binding NTD inhibitor mechanism of the EPI series, if preclinical activities translate to the clinic and sufficient bioavailability is possible, suggests broad scope AR antagonism in prostate cancers expressing escape and/or truncation mutant; however, poor pharmacokinetics of this template suggest that AR overexpression may be difficult to overcome. Phase I dose escalation trials are expected to end in 2022.


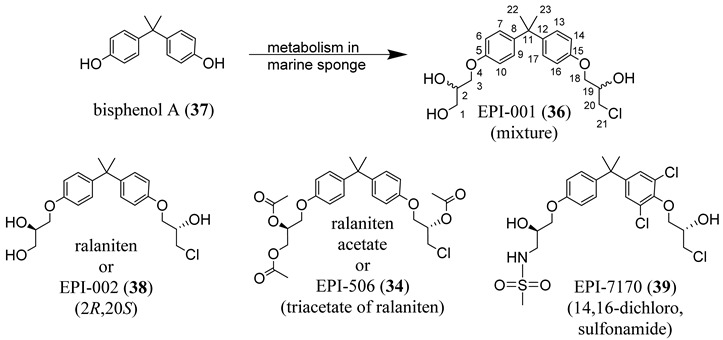


Sadar et al. have also reported chemically unrelated natural products that interact with the NTD. For example, sintokamide A (**40**) was one of the first natural products reported to block the NTD transactivation of the AR in prostate cancer cells [74]. Early preclinical SAR studies of this template were reported recently [75], including **41**. Another agent, niphatenone B (**42**), was isolated from the marine sponge *Niphates digitalis* that represents a novel structural class of AR antagonist. Research found that **41** binds covalently to the AF-1 region of the AR NTD and blocks the proliferation of prostate cancer cells that are dependent on functional AR, and many analogs have been prepared and examined [76].


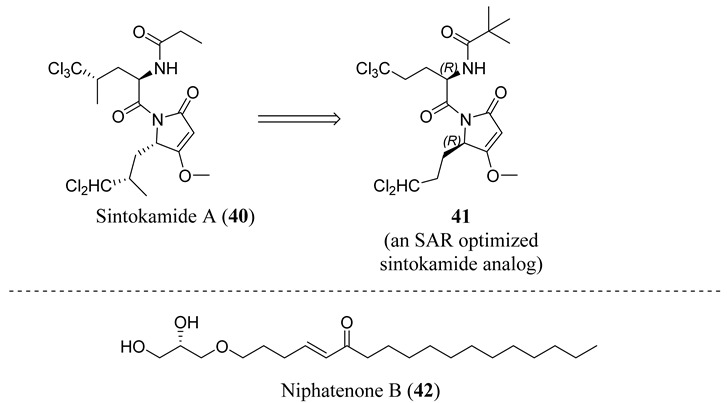


### 4.5. Propanamide AF-1 Inhibitors

A series of NTD-binding compounds emerged from propanamide structure–activity relationship studies. Unlike the structurally similar SARM, enobosarm (**12**), or antiandrogen bicalutamide (**11**), these were full-antagonists with a tertiary amine or nitrogenous B-ring that were discovered to be SARDs. Leads inhibit and degrade a broad scope of expressed ARs to include wtAR, all point mutations tested, and all AR-SVs tested. These molecules bind to the LBD and NTD of AR, but are believed to target AR for degradation via the NTD binding site [67,77], suggesting the ability to overcome not only point mutation resistance, including enzalutamide (**3**) [78] resistance, but also resistance conferred by AR-SV, which is pan-resistant among FDA-approved agents. The initial SARD in this series, UT-69 (43) (78 nM LBD binding; 48 nM inhibition of wtAR), demonstrated poor metabolic stability due to de-methylation of the tertiary amine and hydroxylation of the biaryl B-ring.


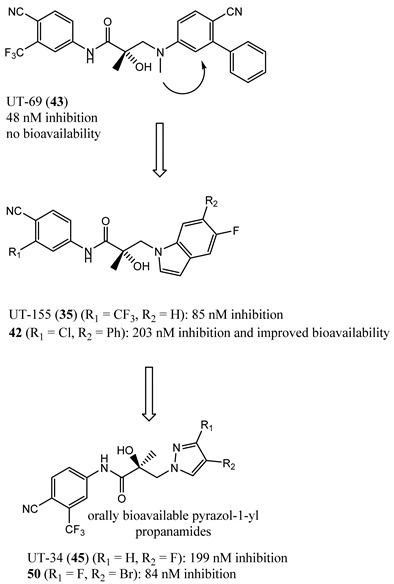


These metabolic liabilities were eliminated by cyclicizing the amine into a series of indoles and indolines [78], exemplified by UT-155 (**35**) (267 nM LBD binding; 85 nM inhibition of wtAR). Despite having reduced AR inhibitory potency, **35** was found to possess improved pharmacokinetic properties such that in vivo activity was observed. Subcutaneously administered **35** reduced tumor weights and degraded full-length AR and AR-SV (AR-V7) in 22RV1 cells, unlike **3**, and further suppressed PSA in LNCaP (T877A) tumors [67].

Biophysical interaction experiments using NTD-only peptides co-incubated with these SARDs and monitored by NMR, fluorescence polarization, and the like [67,77,78], have provided consistent evidence that these compounds bind to an NTD binding site. Unfortunately, the tertiary amine, indole, and indoline templates published thus far had less than exemplary pharmacokinetics.

In order to improve oral bioavailability and reveal the pharmacodynamics possibilities accessible with an NTD-directed SARD, the same research group developed and reported the second generation of these SARDs, which nearly maintained potency but dramatically improved in vivo efficacies [77]. A series of pyrazol-1-yl-propanamide compounds was prepared by introducing the pyrazole moiety as the B-ring in the common A-ring–linkage–B-ring nonsteroidal antiandrogens general pharmacophore. The pyrazoles are exemplified by UT-34 (**45**) (low affinity for LBD (>10 µM in purified LBD); 199 nM inhibition of wtAR), which was the first well-characterized orally bioavailable SARD from this group [77,79]. Unlike previous SARDs, **45** has low binding affinity to LBD but maintained AF-1 binding as supported by steady-state fluorescence emission spectra. Like previous SARDs, **45** requires the tau-5 region of AF-1 for promoting AR degradation through the ubiquitin proteasome pathway to degrade both AR-FL and AR-SVs [77].

SAR studies of these pyrazol-1-yl-propanamides successfully improved potency while retaining oral bioavailability. NTD SARDs were screened in vitro for high potency inhibition of wtAR (IC_50_ in the low nanomolar range, preferably below 50 nM) and high efficacy (>70%) degradation of both AR-FL and AR SV. Similar to the indoles discussed above, these studies demonstrated that the stronger the electron-withdrawing moiety is on the pyrazole ring, the more potent the AR inhibitory activity, with the potency order of 46 (*R^2^* = NO_2_; IC_50_ of 36 nM) > 47 (*R^2^* = CN; 45 nM) > 48 (*R^2^* = CF_3_; 71 nM) > 49 (*R^2^* = Cl; 136 nM) > 45 (*R^2^* = F; 199 nM), but SARD efficacy did not necessary correlate with these AR-FL inhibitory activities (IC_50_). Disubstitution of the pyrazole ring was tolerated without loss of AR inhibitory potency and possibly enhanced bioavailability, as exemplified by 50 (R_1_ = F, R_2_ = Br). Research showed that **50** retained high potency AR inhibition (IC_50_ of 84 nM) and was among the highest efficacy degraders of the pyrazole template with greater than 70% degradation of both AR FL and AR SV at 1 and 10 µM. By comparison, **45** was a less potent wtAR inhibitor (199 nM) but was a full efficacy degrader of AR-FL and AR-SV. We have listed the compounds that are in clinical and preclinical development in Table 1.

## 5. Future Antiandrogen Design and Screening Paradigms

Thus far, all approved antiandrogens are competitive HBP ligands that function by denying access of endogenous androgens to the HBP pocket. As discussed above, this approach seems limited in view of the extreme complexity and highly regulated nature of AR biology and the known resistance mechanisms that these agents elicit. AR agonist activity requires a functional LBD to bind agonist and induce the N/C global conformation, homodimerize, and translocate to the nucleus to recruit coregulatory proteins to either AF-1 or AF-2, and this is further modulated by growth regulatory kinase signaling cascades. The complexity provides multiple points to interfere with AR activity without resorting to blocking HBP binding, and early attempts to explore non-competitive direct-acting AR inhibitors that bind to novel sites of action are discussed above; however, no non-competitive antagonist has been successfully trialed. Further, some LBD (competitive ligands) or NTD-binding AR ligands also have the ability to degrade AR-FL or AR-SV, providing additional advantages in AR axis suppression over traditional canonical antiandrogens. The field of noncanonical inhibitors and SARDs have exerted AR antagonism profiles commensurate in scope to the function of the site blocked [80], but are thus far limited by binding affinity and/or absorption, distribution, metabolism, excretion, and toxicity (ADMET) properties. The molecular structure details of full-length AR in its global agonist and antagonist conformations are not available, and AR biology, in many ways, is still poorly understood. This complexity and still emerging molecular basis of AR biology presents an opportunity for medicinal chemists willing to target nonconventional binding sites. In conclusion, the field of AR-targeted therapeutics to treat advanced PCa is entering a very exciting decade and the patients will see more mechanistically advanced drugs in the market that will provide them extended benefits.

## Figures and Tables

**Table 1:** Summary of compounds in preclinical and clinical development.

Structure/Cmpd ID (Target Activity)	In Vitro Data	In Vivo or Clinical Data
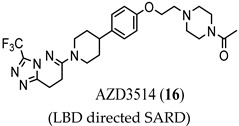 (LBD directed SARD)	Binds AR LBD Downregulates AR	In a phase I clinical trial in CRPC patients, moderate antitumor effects were seen as decreased PSA levels. Trial was discontinued.
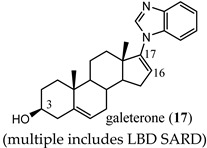 (multiple includes LBD SARD)	Acts as an LBD SARD Acts as a CYP17A1 inhibitor Acts as a competitive antagonist	In a phase III clinical trial in AR-V7-positive mCRPC patients, poor oral bioavailability contributed to poor efficacy. Trial was discontinued.
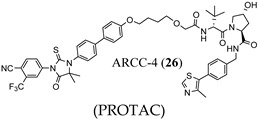 (PROTAC)	Degraded AR in VCaP cells at 5 nM with 98% maximal effect Degraded wtAR and multiple escape mutants	In a phase I clinical trial of ARV110 (structure not shown) in late-stage mCRPC patients, PSA responses were seen at high dose including 2 of 5 escape mutants and 2 of 15 with wtAR. Trial is ongoing.
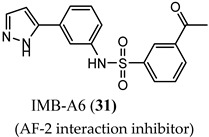 (AF-2 interaction inhibitor)	Blocked PELP-1 binding to the AF-2 binding site at 10 µM	N.R.
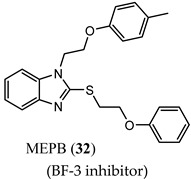 (BF-3 inhibitor)	Binds to BF-3 binding site as determined by X-ray crystallography	Improved symptoms in spinobulbar muscular atrophy (SBMA) mouse model, including ameliorated neuronal loss, neurogenic atrophy, and testicular atrophy. In SBMA model, increased NCoR recruitment to AF-2 binding site. No clinical data.
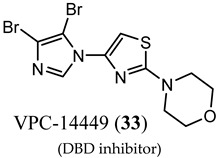 (DBD inhibitor)	Suppressed PSA in LNCaP cells comparably to enzalutamide (3) Inhibited transcriptional activation comparably to 3 Unlike 3, inhibition was abrogated by DBD mutations Suppressed androgen-dependent genes in CRPC model cell lines such as LNCaP, C4-2, AD1, MR49F, 22rv1, and D567	No clinical data.
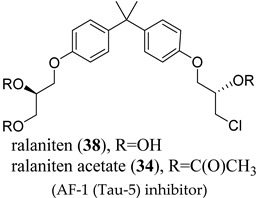 (AF-1 (Tau-5) inhibitor)	For 38: irreversibly bound to NTD, binding is noncompetitive For 38: inhibited androgen-dependent genes in cells with only AR-SV (v567es; i.e., no AR FL)	For 38: in VCaP tumors, suppressed AR-V7-dependent genes. For 34: in a phase I clinical trial in patients that previously failed enzalutamide or abiraterone treatment, <30% decreased PSA at very large doses up to 3.6 g. Trial discontinued.
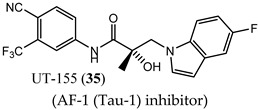 (AF-1 (Tau-1) inhibitor)	Inhibited wtAR transcriptional activation (85 nM) Reversibly bound to purified LBD (267 nM) Completely degraded AR FL and AR-V7 (100%/100%) NTD binding via multiple biophysical measurements	SQ administration decreased tumor weights and degraded AR FL and AR-V7 intratumor. No oral bioavailability. No clinical data.
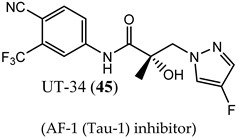 (AF-1 (Tau-1) inhibitor)	Inhibited wtAR transcriptional activation (199 nM) Weak reversible purified LBD binding (>10 µM) Completely degraded AR FL and AR-V7 (100%/100%) NTD binding	Unprecedented full regression with PO administration in intact rats with MR49F LNCaP and enzalutamide-resistant VCaP xenografts. No clinical data.
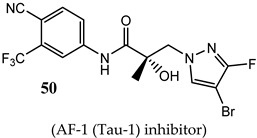 (AF-1 (Tau-1) inhibitor)	Inhibited wtAR transcriptional activation (84 nM) High efficacy degradation of AR-FL and AR-V7 (70%/70%)	80% tumor growth inhibition with PO administration in intact rats with xenografts derived from the enzalutamide-resistant (Enz-R) VCaP cell line. No clinical data.

## Abbreviations

ADT: androgen deprivation therapy; ADMET: absorption, distribution, metabolism, excretion, and toxicity; AF-1: activating function-1; AF-2: activating function-2; AR: androgen receptor; AR-FL: full-length androgen receptor; ARE: androgen responsive element; AR-SV: splice variant androgen receptor; BF-3: binding function-3; CRBN: cereblon; CRPC: castration-resistant prostate cancer; CYP17A1: cytochrome P450 17A1, also called steroid 17α-hydroxylase and 17,20-lyase; DBD: DNA binding domain; DES: diethylstilbestrol; DHT: 5α-dihydrotestosterone; DNA: deoxyribonucleic acid; FL: full-length; GR: glucocorticoid receptor; FDA: Food and Drug Administration; HBP: hormone binding pocket; LBD: ligand binding domain; mCRPC: metastatic castration-resistant prostate cancer; MDVR: enzalutamide (MDV3100)-resistant; mtAR: mutant androgen receptor; nmCRPC: nonmetastatic castration-resistant prostate cancer; NMR: nuclear magnetic resonance; NCoR: nuclear receptor corepressor; NTD: N-terminal domain; OS: overall survival; PCa: prostate cancer; PDB: Protein Database; PEG: polyethylene glycol; PELP-1: proline-, glutamic acid-, and leucine-rich peptide-1; PK: pharmacokinetic; PR: progesterone receptor; PROTAC: proteolysis-targeting chimera; PSA: prostate-specific antigen; SAR: structure–activity relationship; SARD: selective androgen receptor degrader (or downregulator); SARM: selective androgen receptor modulator; SBMA: spinobulbar muscular atrophy; TAU: transcription activation unit; Tau-1: transcription activation unit-1; Tau-2: transcription activation unit-2; TMPRSS2–ERG: a fusion of TMPRSS2 (transmembrane protease, serine 2) to ETS-related gene (ERG); VHL: Von Hippel–Lindau; Ub: ubiquitin; wtAR: wild-type androgen receptor; C4-2: prostate cancer cell line derived from LNCaP; C4-2B: prostate cancer cell line derived from LNCaP; CWR22Rv1 (22Rv1): AR- and AR splice variant-positive prostate cancer cells; DU145: AR-negative prostate cancer cells; LNCaP: lymph node metastasis AR-positive prostate cancer cell line; MR49C: enzalutamide-resistant LNCaP; MR49F: enzalutamide-resistant LNCaP; VCaP: AR-positive prostate cancer cell line.

## References

[1] Siegel R.L., Miller K.D., Jemal A. (2020). Cancer statistics, 2020. CA Cancer J. Clin..

[2] Shah R.B., Mehra R., Chinnaiyan A.M., Shen R., Ghosh D., Zhou M., Macvicar G.R., Varambally S., Harwood J., Bismar T.A. (2004). Androgen-independent prostate cancer is a heterogeneous group of diseases: Lessons from a rapid autopsy program. Cancer Res..

[3] Brinkmann A.O., Faber P.W., van Rooij H.C., Kuiper G.G., Ris C., Klaassen P., van der Korput J.A., Voorhorst M.M., van Laar J.H., Mulder E. (1989). The human androgen receptor: Domain structure, genomic organization and regulation of expression. J. Steroid Biochem..

[4] Taylor B.S., Schultz N., Hieronymus H., Gopalan A., Xiao Y., Carver B.S., Arora V.K., Kaushik P., Cerami E., Reva B. (2010). Integrative genomic profiling of human prostate cancer. Cancer Cell.

[5] Huggins C. (1942). Effect of Orchiectomy and Irradiation on Cancer of the Prostate. Ann. Surg..

[6] Bluemn E.G., Coleman I.M., Lucas J.M., Coleman R.T., Hernandez-Lopez S., Tharakan R., Bianchi-Frias D., Dumpit R.F., Kaipainen A., Corella A.N. (2017). Androgen Receptor Pathway-Independent Prostate Cancer Is Sustained through FGF Signaling. Cancer Cell.

[7] Epstein J.I., Amin M.B., Beltran H., Lotan T.L., Mosquera J.M., Reuter V.E., Robinson B.D., Troncoso P., Rubin M.A. (2014). Proposed morphologic classification of prostate cancer with neuroendocrine differentiation. Am. J. Surg. Pathol..

[8] Wadosky K.M., Koochekpour S. (2017). Androgen receptor splice variants and prostate cancer: From bench to bedside. Oncotarget.

[9] Brand L.J., Dehm S.M. (2013). Androgen receptor gene rearrangements: New perspectives on prostate cancer progression. Curr. Drug Targets.

[10] Xu J., Qiu Y. (2016). Role of androgen receptor splice variants in prostate cancer metastasis. Asian J. Urol..

[11] Melnyk J.E., Steri V., Nguyen H.G., Hann B., Feng F.Y., Shokat K.M. (2020). The splicing modulator sulfonamide indisulam reduces AR-V7 in prostate cancer cells. Bioorg. Med. Chem..

[12] Armstrong A.J., Luo J., Nanus D.M., Giannakakou P., Szmulewitz R.Z., Danila D.C., Healy P., Anand M., Berry W.R., Zhang T. (2020). Prospective Multicenter Study of Circulating Tumor Cell AR-V7 and Taxane Versus Hormonal Treatment Outcomes in Metastatic Castration-Resistant Prostate Cancer. Jco. Precis. Oncol..

[13] Liu C., Armstrong C., Zhu Y., Lou W., Gao A.C. (2016). Niclosamide enhances abiraterone treatment via inhibition of androgen receptor variants in castration resistant prostate cancer. Oncotarget.

[14] Liu C., Lou W., Zhu Y., Nadiminty N., Schwartz C.T., Evans C.P., Gao A.C. (2014). Niclosamide inhibits androgen receptor variants expression and overcomes enzalutamide resistance in castration-resistant prostate cancer. Clin. Cancer Res..

[15] Mainwaring W.I. (1970). The separation of androgen receptor and 5 alpha-reductase activities in subcellular fractions of rat prostate. Biochem. Biophys. Res. Commun..

[16] Sack J.S., Kish K.F., Wang C., Attar R.M., Kiefer S.E., An Y., Wu G.Y., Scheffler J.E., Salvati M.E., Krystek S.R. (2001). Crystallographic structures of the ligand-binding domains of the androgen receptor and its T877A mutant complexed with the natural agonist dihydrotestosterone. Proc. Natl. Acad. Sci. USA.

[17] Jacobo E., Schmidt J.D., Weinstein S.H., Flocks R.H. (1976). Comparison of flutamide (SCH-13521) and diethylstilbestrol in untreated advanced prostatic cancer. Urology.

[18] Furr B.J., Valcaccia B., Curry B., Woodburn J.R., Chesterson G., Tucker H. (1987). ICI 176,334: A novel non-steroidal, peripherally selective antiandrogen. J. Endocrinol..

[19] Dalton J.T., Mukherjee A., Zhu Z., Kirkovsky L., Miller D.D. (1998). Discovery of nonsteroidal androgens. Biochem. Biophys. Res. Commun..

[20] Yin D., Gao W., Kearbey J.D., Xu H., Chung K., He Y., Marhefka C.A., Veverka K.A., Miller D.D., Dalton J.T. (2003). Pharmacodynamics of selective androgen receptor modulators. J. Pharm. Exp..

[21] Marhefka C.A., Gao W., Chung K., Kim J., He Y., Yin D., Bohl C., Dalton J.T., Miller D.D. (2004). Design, synthesis, and biological characterization of metabolically stable selective androgen receptor modulators. J. Med. Chem..

[22] Solomon Z.J., Mirabal J.R., Mazur D.J., Kohn T.P., Lipshultz L.I., Pastuszak A.W. (2019). Selective Androgen Receptor Modulators: Current Knowledge and Clinical Applications. Sex. Med. Rev..

[23] Hsu C.L., Liu J.S., Wu P.L., Guan H.H., Chen Y.L., Lin A.C., Ting H.J., Pang S.T., Yeh S.D., Ma W.L. (2014). Identification of a new androgen receptor (AR) co-regulator BUD31 and related peptides to suppress wild-type and mutated AR-mediated prostate cancer growth via peptide screening and X-ray structure analysis. Mol. Oncol..

[24] Aikawa K., Asano M., Ono K., Habuka N., Yano J., Wilson K., Fujita H., Kandori H., Hara T., Morimoto M. (2017). Synthesis and biological evaluation of novel selective androgen receptor modulators (SARMs) Part III: Discovery of 4-(5-oxopyrrolidine-1-yl) benzonitrile derivative 2f as a clinical candidate. Bioorg. Med. Chem..

[25] Bohl C.E., Miller D.D., Chen J., Bell C.E., Dalton J.T. (2005). Structural basis for accommodation of nonsteroidal ligands in the androgen receptor. J. Biol. Chem..

[26] Saeed A., Vaught G.M., Gavardinas K., Matthews D., Green J.E., Losada P.G., Bullock H.A., Calvert N.A., Patel N.J., Sweetana S.A. (2016). 2-Chloro-4-[[(1R,2R)-2-hydroxy-2-methyl-cyclopentyl]amino]-3-methyl-benzonitrile: A Transdermal Selective Androgen Receptor Modulator (SARM) for Muscle Atrophy. J. Med. Chem..

[27] Zhang Z., Connolly P.J., Lim H.K., Pande V., Meerpoel L., Teleha C., Branch J.R., Ondrus J., Hickson I., Bush T. (2021). Discovery of JNJ-63576253: A Clinical Stage Androgen Receptor Antagonist for F877L Mutant and Wild-Type Castration-Resistant Prostate Cancer (mCRPC). J. Med. Chem..

[28] Ge R., Xu X., Xu P., Li L., Li Z., Bian J. (2018). Degradation of Androgen Receptor through Small Molecules for Prostate Cancer. Curr. Cancer Drug Targets.

[29] Wang R., Lin W., Lin C., Li L., Sun Y., Chang C. (2016). ASC-J9((R)) suppresses castration resistant prostate cancer progression via degrading the enzalutamide-induced androgen receptor mutant AR-F876L. Cancer Lett..

[30] Omlin A., Jones R.J., van der Noll R., Satoh T., Niwakawa M., Smith S.A., Graham J., Ong M., Finkelman R.D., Schellens J.H. (2015). AZD3514, an oral selective androgen receptor down-regulator in patients with castration-resistant prostate cancer—Results of two parallel first-in-human phase I studies. Investig. New Drugs.

[31] Latysheva A.S., Zolottsev V.A., Veselovsky A.V., Scherbakov K.A., Morozevich G.E., Pokrovsky V.S., Novikov R.A., Timofeev V.P., Tkachev Y.V., Misharin A.Y. (2020). New steroidal oxazolines, benzoxazoles and benzimidazoles related to abiraterone and galeterone. Steroids.

[32] Njar V.C., Brodie A.M. (2015). Discovery and development of Galeterone (TOK-001 or VN/124–1) for the treatment of all stages of prostate cancer. J. Med. Chem..

[33] Alyamani M., Li Z., Berk M., Li J., Tang J., Upadhyay S., Auchus R.J., Sharifi N. (2017). Steroidogenic Metabolism of Galeterone Reveals a Diversity of Biochemical Activities. Cell Chem. Biol..

[34] Yu Z., Cai C., Gao S., Simon N.I., Shen H.C., Balk S.P. (2014). Galeterone prevents androgen receptor binding to chromatin and enhances degradation of mutant androgen receptor. Clin. Cancer Res..

[35] Kwegyir-Afful A.K., Ramalingam S., Purushottamachar P., Ramamurthy V.P., Njar V.C. (2015). Galeterone and VNPT55 induce proteasomal degradation of AR/AR-V7, induce significant apoptosis via cytochrome c release and suppress growth of castration resistant prostate cancer xenografts in vivo. Oncotarget.

[36] Kwegyir-Afful A.K., Ramalingam S., Ramamurthy V.P., Purushottamachar P., Murigi F.N., Vasaitis T.S., Huang W., Kane M.A., Zhang Y., Ambulos N. (2019). Galeterone and The Next Generation Galeterone Analogs, VNPP414 and VNPP433–3beta Exert Potent Therapeutic Effects in Castration-/Drug-Resistant Prostate Cancer Preclinical Models In Vitro and In Vivo. Cancers (Basel.).

[37] Sakamoto K.M., Kim K.B., Kumagai A., Mercurio F., Crews C.M., Deshaies R.J. (2001). Protacs: Chimeric molecules that target proteins to the Skp1-Cullin-F box complex for ubiquitination and degradation. Proc. Natl. Acad. Sci. USA.

[38] Schneekloth A.R., Pucheault M., Tae H.S., Crews C.M. (2008). Targeted intracellular protein degradation induced by a small molecule: En route to chemical proteomics. Bioorg. Med. Chem. Lett..

[39] Lohbeck J., Miller A.K. (2016). Practical synthesis of a phthalimide-based Cereblon ligand to enable PROTAC development. Bioorg. Med. Chem. Lett..

[40] Han X., Wang C., Qin C., Xiang W., Fernandez-Salas E., Yang C.Y., Wang M., Zhao L., Xu T., Chinnaswamy K. (2019). Discovery of ARD-69 as a Highly Potent Proteolysis Targeting Chimera (PROTAC) Degrader of Androgen Receptor (AR) for the Treatment of Prostate Cancer. J. Med. Chem..

[41] Guo C., Linton A., Kephart S., Ornelas M., Pairish M., Gonzalez J., Greasley S., Nagata A., Burke B.J., Edwards M. (2011). Discovery of aryloxy tetramethylcyclobutanes as novel androgen receptor antagonists. J. Med. Chem..

[42] Zhao L., Han X., Lu J., McEachern D., Wang S. (2020). A highly potent PROTAC androgen receptor (AR) degrader ARD-61 effectively inhibits AR-positive breast cancer cell growth in vitro and tumor growth in vivo. Neoplasia.

[43] Shibata N., Nagai K., Morita Y., Ujikawa O., Ohoka N., Hattori T., Koyama R., Sano O., Imaeda Y., Nara H. (2018). Development of Protein Degradation Inducers of Androgen Receptor by Conjugation of Androgen Receptor Ligands and Inhibitor of Apoptosis Protein Ligands. J. Med. Chem..

[44] Scott D.E., Rooney T.P.C., Bayle E.D., Mirza T., Willems H.M.G., Clarke J.H., Andrews S.P., Skidmore J. (2020). Systematic Investigation of the Permeability of Androgen Receptor PROTACs. ACS Med. Chem. Lett..

[45] Flanagan J.J., Neklesa T.K. (2019). Targeting Nuclear Receptors with PROTAC degraders. Mol. Cell. Endocrinol..

[46] Beretta G.L., Zaffaroni N. (2019). Androgen Receptor-Directed Molecular Conjugates for Targeting Prostate Cancer. Front. Chem..

[47] Balbas M.D., Evans M.J., Hosfield D.J., Wongvipat J., Arora V.K., Watson P.A., Chen Y., Greene G.L., Shen Y., Sawyers C.L. (2013). Overcoming mutation-based resistance to antiandrogens with rational drug design. Elife.

[48] Hara T., Miyazaki J., Araki H., Yamaoka M., Kanzaki N., Kusaka M., Miyamoto M. (2003). Novel mutations of androgen receptor: A possible mechanism of bicalutamide withdrawal syndrome. Cancer Res..

[49] Liu H., An X., Li S., Wang Y., Li J., Liu H. (2015). Interaction mechanism exploration of R-bicalutamide/S-1 with WT/W741L AR using molecular dynamics simulations. Mol. Biosyst..

[50] Fenton M.A., Shuster T.D., Fertig A.M., Taplin M.E., Kolvenbag G., Bubley G.J., Balk S.P. (1997). Functional characterization of mutant androgen receptors from androgen-independent prostate cancer. Clin. Cancer Res..

[51] Tan J., Hamil Y.S.K.G., Gregory C.W., Zang D.Y., Sar M., Gumerlock P.H., White R.W.D., Pretlow T.G., Harris S.E., Wilson E.M. (1997). Dehydroepiandrosterone activates mutant androgen receptors expressed in the androgen-dependent human prostate cancer xenograft CWR22 and LNCaP cells. Mol. Endocrinol..

[52] (2000). Glucocorticoids can promote androgen-independent growth of prostate cancer cells through a mutated androgen receptor. Nat. Med..

[53] Korpal M., Korn J.M., Gao X., Rakiec D.P., Ruddy D.A., Doshi S., Yuan J., Kovats S.G., Kim S., Cooke V.G. (2013). An F876L mutation in androgen receptor confers genetic and phenotypic resistance to MDV3100 (enzalutamide). Cancer Discov..

[54] Fujii S., Kagechika H. (2019). Androgen receptor modulators: A review of recent patents and reports (2012–2018). Expert Opin. Pat..

[55] Li H., Ban F., Dalal K., Leblanc E., Frewin K., Ma D., Adomat H., Rennie P.S., Cherkasov A. (2014). Discovery of small-molecule inhibitors selectively targeting the DNA-binding domain of the human androgen receptor. J. Med. Chem..

[56] Nadal M., Prekovic S., Gallastegui N., Helsen C., Abella M., Zielinska K., Gay M., Vilaseca M., Taules M., Houtsmuller A.B. (2017). Structure of the homodimeric androgen receptor ligand-binding domain. Nat. Commun..

[57] Dalal K., Ban F., Li H., Morin H., Roshan-Moniri M., Tam K.J., Shepherd A., Sharma A., Peacock J., Carlson M.L. (2018). Selectively targeting the dimerization interface of human androgen receptor with small-molecules to treat castration-resistant prostate cancer. Cancer Lett..

[58] Munuganti R.S., Leblanc E., Axerio-Cilies P., Labriere C., Frewin K., Singh K., Hassona M.D., Lack N.A., Li H., Ban F. (2013). Targeting the binding function 3 (BF3) site of the androgen receptor through virtual screening. 2. development of 2-((2-phenoxyethyl) thio)-1H-benzimidazole derivatives. J. Med. Chem..

[59] Liu Y., Wu M., Wang T., Xie Y., Cui X., He L., He Y., Li X., Liu M., Hu L. (2018). Structural Based Screening of Antiandrogen Targeting Activation Function-2 Binding Site. Front. Pharm..

[60] Lack N.A., Axerio-Cilies P., Tavassoli P., Han F.Q., Chan K.H., Feau C., LeBlanc E., Guns E.T., Guy R.K., Rennie P.S. (2011). Targeting the binding function 3 (BF3) site of the human androgen receptor through virtual screening. J. Med. Chem..

[61] Badders N.M., Korff A., Miranda H.C., Vuppala P.K., Smith R.B., Winborn B.J., Quemin E.R., Sopher B.L., Dearman J., Messing J. (2018). Selective modulation of the androgen receptor AF2 domain rescues degeneration in spinal bulbar muscular atrophy. Nat. Med..

[62] Dalal K., Roshan-Moniri M., Sharma A., Li H., Ban F., Hassona M.D., Hsing M., Singh K., LeBlanc E., Dehm S. (2014). Selectively targeting the DNA-binding domain of the androgen receptor as a prospective therapy for prostate cancer. J. Biol. Chem..

[63] Dalal K., Che M., Que N.S., Sharma A., Yang R., Lallous N., Borgmann H., Ozistanbullu D., Tse R., Ban F. (2017). Bypassing Drug Resistance Mechanisms of Prostate Cancer with Small Molecules that Target Androgen Receptor-Chromatin Interactions. Mol. Cancer.

[64] Messner E.A., Steele T.M., Tsamouri M.M., Hejazi N., Gao A.C., Mudryj M., Ghosh P.M. (2020). The Androgen Receptor in Prostate Cancer: Effect of Structure, Ligands and Spliced Variants on Therapy. Biomedicines.

[65] Jenster G., van der Korput H.A., Trapman J., Brinkmann A.O. (1995). Identification of two transcription activation units in the N-terminal domain of the human androgen receptor. J. Biol. Chem..

[66] De Mol E., Fenwick R.B., Phang C.T., Buzon V., Szulc E., de la Fuente A., Escobedo A., Garcia J., Bertoncini C.W., Estebanez-Perpina E. (2016). EPI-001, A Compound Active against Castration-Resistant Prostate Cancer, Targets Transactivation Unit 5 of the Androgen Receptor. ACS Chem. Biol..

[67] Ponnusamy S., Coss C.C., Thiyagarajan T., Watts K., Hwang D.J., He Y., Selth L.A., McEwan I.J., Duke C.B., Pagadala J. (2017). Novel Selective Agents for the Degradation of Androgen Receptor Variants to Treat Castration-Resistant Prostate Cancer. Cancer Res..

[68] Jenster G., van der Korput H.A., van Vroonhoven C., van der Kwast T.H., Trapman J., Brinkmann A.O. (1991). Domains of the human androgen receptor involved in steroid binding, transcriptional activation, and subcellular localization. Mol. Endocrinol..

[69] McEwan I.J. (2012). Intrinsic disorder in the androgen receptor: Identification, characterisation and drugability. Mol. Biosyst..

[70] Andersen R.J., Mawji N.R., Wang J., Wang G., Haile S., Myung J.K., Watt K., Tam T., Yang Y.C., Banuelos C.A. (2010). Regression of castrate-recurrent prostate cancer by a small-molecule inhibitor of the amino-terminus domain of the androgen receptor. Cancer Cell.

[71] Myung J.K., Banuelos C.A., Fernandez J.G., Mawji N.R., Wang J., Tien A.H., Yang Y.C., Tavakoli I., Haile S., Watt K. (2013). An androgen receptor N-terminal domain antagonist for treating prostate cancer. J. Clin. Investig..

[72] Hu R., Lu C., Mostaghel E.A., Yegnasubramanian S., Gurel M., Tannahill C., Edwards J., Isaacs W.B., Nelson P.S., Bluemn E. (2012). Distinct transcriptional programs mediated by the ligand-dependent full-length androgen receptor and its splice variants in castration-resistant prostate cancer. Cancer Res..

[73] Hirayama Y., Tam T., Jian K., Andersen R.J., Sadar M.D. (2020). Combination therapy with androgen receptor N-terminal domain antagonist EPI-7170 and enzalutamide yields synergistic activity in AR-V7-positive prostate cancer. Mol. Oncol..

[74] Banuelos C.A., Tavakoli I., Tien A.H., Caley D.P., Mawji N.R., Li Z., Wang J., Yang Y.C., Imamura Y., Yan L. (2016). Sintokamide A Is a Novel Antagonist of Androgen Receptor That Uniquely Binds Activation Function-1 in Its Amino-terminal Domain. J. Biol. Chem..

[75] Yan L., Banuelos C.A., Mawji N.R., Patrick B.O., Sadar M.D., Andersen R.J. (2020). Structure-Activity Relationships for the Marine Natural Product Sintokamides: Androgen Receptor N-Terminus Antagonists of Interest for Treatment of Metastatic Castration-Resistant Prostate Cancer. J. Nat. Prod..

[76] Banuelos C.A., Lal A., Tien A.H., Shah N., Yang Y.C., Mawji N.R., Meimetis L.G., Park J., Kunzhong J., Andersen R.J. (2014). Characterization of niphatenones that inhibit androgen receptor N-terminal domain. PLoS ONE.

[77] Ponnusamy S., He Y., Hwang D.J., Thiyagarajan T., Houtman R., Bocharova V., Sumpter B.G., Fernandez E., Johnson D.L., Du Z. (2019). Orally-Bioavailable Androgen Receptor Degrader, A Potential Next-Generation Therapeutic for Enzalutamide-Resistant Prostate Cancer. Clin. Cancer Res..

[78] Hwang D.J., He Y., Ponnusamy S., Mohler M.L., Thiyagarajan T., McEwan I.J., Narayanan R., Miller D.D. (2019). New Generation of Selective Androgen Receptor Degraders: Our Initial Design, Synthesis, and Biological Evaluation of New Compounds with Enzalutamide-Resistant Prostate Cancer Activity. J. Med. Chem..

[79] He Y., Hwang D.J., Ponnusamy S., Thiyagarajan T., Mohler M.L., Narayanan R., Miller D.D. (2020). Pyrazol-1-yl-propanamides as SARD and Pan-Antagonists for the Treatment of Enzalutamide-Resistant Prostate Cancer. J. Med. Chem..

[80] Haendler B., Cleve A. (2012). Recent developments in antiandrogens and selective androgen receptor modulators. Mol. Cell. Endocrinol..

